# Supplementary material for: Novel and optimized mouse behavior enabled by fully autonomous HABITS: Home-cage assisted behavioral innovation and testing system
Source: eLife. 2025 Sep 16;14:RP104833. doi: 10.7554/eLife.104833 (PMC12440354; doi:10.7554/eLife.104833)
Supplement: Supplementary file 1. [file elife-104833-supp1.docx]

**Supplementary File 1. Building materials of HABITS.**

| **Parts** | **Quantity** | **Model and Supplier** | **Price*** |
| --- | --- | --- | --- |
| Acrylic board | N/A | Custom | $ 8.47 |
| 3D printing | N/A | Sogaworks.com | $ 9.88 |
| Microcontroller | 1 | Teensy 3.6, PJRC Inc. | $ 36.43 |
| SD card | 1 | SDQUNC-16GB, SanDisk Inc. | $ 3.94 |
| Load cell and driver module | 1 | YZC191 and HX711, Guangce.com | $ 3.57 |
| Licking sensor module | 1 | MPR121, Taobao.com | $ 0.49 |
| Wi-Fi module | 1 | ESP8266, Espressif Inc. | $ 0.76 |
| LED | 3 | GS5050RGB and GS3528UWC, Guangsheng Inc. | $ 0.14 |
| Buzzer | 3 | PS1740P02, TDK Inc. | $ 1.27 |
| Solid State Relay | 2 | SDD-5HB, XYF Inc. | $ 9.32 |
| Peristaltic Pump | 2 | KPP-DCL-S01W, kamoer Inc. | $ 20.0 |
| Power Adapter | 1 | ABLK-T1201, Aobaolike Inc. | $ 1.40 |
| Others | N/A | N/A | $ 3.07 |
| **Total** |  |  | $ 98.74 |

*Price was originally in Chinese yuan and converted to US dollar with exchange rate of 7:1.
