# Supplementary material for: Novel and optimized mouse behavior enabled by fully autonomous HABITS: Home-cage assisted behavioral innovation and testing system
Source: eLife. 2025 Sep 16;14:RP104833. doi: 10.7554/eLife.104833 (PMC12440354; doi:10.7554/eLife.104833)
Supplement: Supplementary file 2. [file elife-104833-supp2.docx]

**Supplementary File 2**

**Tasks tested in HABITS**

*Value-based dynamic foraging task*

In Figure 2-figure supplement 3, we introduced the dynamic foraging task. Mice must first lick the center spout to initiate a trial block. Each block consists of one or more trials, and if a mouse fails to make a choice in any given trial, the block ends and waits for the mouse to trigger the next one. Within a trial block, trials are separated by an inter-trial interval (ITI) ranging randomly from 0.5 to 2 seconds, with an average of 1 second. The next trial automatically begins after the ITI.

At the start of each trial, a 500ms light cue is emitted from the top LED, signaling the beginning of the trial. This is followed by a delay period lasting either 1 or 1.5 seconds, during which the mouse must restrict from licking the left or right spouts. If the mouse licks prematurely, the trial pauses for 300ms. After delay, a 6 kHz pure tone lasting up to 2000ms is played from the top buzzer, prompting the mouse to choose by licking either the left or right spout. The first lick during this period is registered as choice for this trial, followed by an auditory cue lasting 50ms (3 kHz for left, 10 kHz for right). If the choice matches the assigned reward direction, a 0.25μL water reward is dispensed from the corresponding spout.

In this task, mice are unable to obtain any useful cues from single trial to make a rewarded choice. All rewards in individual trials are randomly assigned to the left or right spouts according to default probabilities. We use a minimum of 500 trials per rotation, during which the reward probabilities for left and right spouts are rotated in the following sequence: {60:10}, {52.5:17.5}, {10:60}, and {17.5:52.5}. Additionally, mice cannot obtain rewards from one spout if the reward allocated to the other spout has not yet been consumed. Only when the mouse chooses the spout with the higher reward probability in more than 50% probabilities, the rotation advances. As the mouse's performance hits criteria, the frequency of rotations increases, starting from minimum every 500 trials and gradually decreasing to every 100 trials.

*Contingency reversal learning*

In Figure 3A, we extended the self-initiated paradigm (as shown in Figure 2-figure supplement 2) by introducing multiple reversals in the relationship between stimulus and action. Specifically, when a mouse achieves an accuracy rate of over 75% in the most recent 100 trials, the association between sound frequency and reward direction is immediately reversed, while the trial structure and parameters remain unchanged. The mouse receives no additional cues indicating the rule reversal, relying solely on reward and error feedback to adapt. Subsequently, the contingency is reversed each time the mouse reaches the specified accuracy under the current rule.

*Working Memory Task*

In Figure 3B, the mouse begins by licking the central spout to initiate a trial. After a brief delay, the first stimulus is presented, lasting 500ms, and consisting of a sequence of regular clicks. This is followed by a 200ms delay period, during which the mouse must refrain from licking the side spouts. Any licking during this delay immediately terminates the trial and results in a noise burst and a timeout penalty. Following the delay, a second 500ms stimulus of regular clicks is presented. The mouse must then wait for a brief response cue after the delay and indicate its choice by licking either side spout, receiving feedback based on its decision: correct choices yield a water reward, while incorrect choices trigger a 500ms noise burst and a timeout. The correct choice is determined by comparing the click rates of the two stimuli—when the first stimulus has a higher click rate, the reward is on the right side, and when it is lower, the reward is on the left.

The trial types are generated using an anti-bias algorithm to determine the left or right reward direction, after which one of the four possible click rates for that direction is randomly selected as the stimulus for the current trial. The SGM comprises five click rates separated by octave intervals: 8, 16, 32, 64, and 128 Hz.

During the testing phase, the delay period is randomly sampled between 500 and 2000ms. Additionally, in half of the trials, the click rate of the second stimulus is fixed at 32 Hz, while the click rate of the first stimulus is randomly sampled from the set {16, 20, 25, 32, 40, 50, 64 Hz}. If the first click rate is set at 32 Hz, the rewarded choice is randomly assigned to the left or right spout.

In Figure 3-figure supplement 1, a 3x3 SGM was used with a two-octave difference between the click rates of the first and second stimuli. After training, a certain percentage of probe trials were introduced, where the click rates of the two stimuli were identical, and the reward direction was randomly assigned. The mouse's performance in these probe trials was at chance level, indicating that the mouse indeed relied on comparing the frequencies of the two stimuli to complete the task.

*Evidence accumulation*

In Figure 3C, a new trial block is initiated when a mouse licks either of the two spouts. After a brief delay (200-500ms), independent click sequences, following an exponential distribution with predefined parameters, are generated from speakers on both sides. After a second brief delay period, a response cue signals the mouse to choose by licking either the left or right spout. The mouse should select the left spout if the click rate on the left is higher, and the right spout if the click rate on the right is higher. Correct choices are immediately rewarded with 0.25 µl of water at the corresponding spout, while incorrect choices result in a noise burst and a timeout penalty.

The 1000ms sample period is divided into 40 bins, each lasting 25ms. To approximate a discrete event sequence with an exponential distribution, a coin-flip method is used for each bin to determine whether a click event occurs, based on the set probability. Each click event consists of a 10ms, 10 kHz auditory cue followed by a 15ms interval. The hazard rates for generating clicks on the left and right sides are randomly selected from {39:1, 37:3, 31:9, 26:14}. The onset time of the clicks is randomly chosen from within the first 0 to 900ms of the sample period, in 100ms increments.

*Evidence accumulation with multimodal integration*

In Figure 3D, a trial block is initiated when the mouse licks either the left or right spout. After a brief delay, a top speaker emits click sequences following an exponential distribution, while both the left and right white LEDs flash according to the same event sequence. Following the delay period, a response cue indicates the start of the response period, during which the mouse must decide if the event rate exceeds 12 clicks per second. If the event rate is below 12, the mouse should choose the left spout; if it is above 12, the right spout should be chosen. For trials where the event rate is exactly 12, the reward is randomly assigned to either the left or right spout.

The sample period is divided into 40 bins, each lasting 40ms. Similar to the previous task, a coin-flip method is used to approximate the exponential distribution of discrete event sequences, determining whether a multimodal event occurs in each bin. Each multimodal event consists of 20ms light flash paired with an auditory click, followed by 20ms interval. For trials where the reward is assigned to the left spout, the hazard rate for event sequence generation is 4; for trials with the reward on the right spout, the hazard rate is 20. The sample period lasts for 1600ms, with the event onset fixed at 600ms to ensure that the events continue for 1000ms.

During the testing phase, a portion of the trials had event onset times randomly selected from 0, 400, 800, or 1200ms after the start. We also tested the mouse’s performance under unimodal conditions, using only clicks or only flashes as stimuli. To verify the effect of multimodal integration, we adjusted the sound decibel level (by controlling PWM duty cycle) for clicks and the brightness (by controlling PWM amplitude) for flashes to achieve similar performance levels under unimodal conditions. We then examined whether there was an enhancement effect under multimodal stimulation with these parameter settings.

*Confidence-proved task*

In Figure 3E, mice initiated each trial by licking the central spout, while suppressing licking of the lateral spouts for a brief period. Following this, a sound stimulus ranging from 8 kHz to 32 kHz was emitted from the top buzzer for a duration of up to 1000ms. During the first 250ms of the stimulus, the mice had to inhibit any early licks. Failure to do so triggered 100ms noise cue, prematurely terminated the current trial, and imposed a timeout penalty lasting 3 to 7 seconds. The mice's first choice of either the left or right spout after the 250ms sample period was recorded as their decision, ending the stimulus. If the choice was correct, a small reward (approximately 0.25μL) was delivered at the corresponding spout after a brief delay. The mice then had to confirm their decision by licking the rewarded spout within 1000ms to receive a second reward (0.25μL); failure to confirm was treated as a no-response trial. During the delay, mice were required to maintain their choice, refraining from licking any other spout. Licking a non-chosen spout during the delay was interpreted as forfeiture of the decision, resulting in the termination of the trial. If the central spout was licked during this period, the trial ended immediately and a new trial began. For correct choices, the delay duration was sampled from an exponential distribution with a maximum of 5000ms, a minimum of 500ms, and an average of 1000ms. For incorrect choices, the delay was fixed at 20 seconds, with no additional error feedback provided; mice had to lick the other spouts twice to terminate the trial. Trials where mice did not actively terminate within 20 seconds were classified as no-response trials. During the sample period, the task was to discriminate whether the sound frequency exceeded 16 kHz, indicating a right spout choice, or was below 16 kHz, indicating a left spout choice. The frequency range was logarithmically partitioned around the 16 kHz midpoint into nine intervals: {-1, -0.6, -0.2, -0.1, 0, 0.1, 0.2, 0.6, 1}. Negative values corresponded to a left choice, positive values to a right choice, and 0 represented a random choice between left and right. During the testing phase, a certain percentage of the trials were designated as probe trials: when the mice made a correct choice, the delay was set to 20 seconds, and the first reward was omitted. The mice were required to lick another spout to terminate these trials.

*Continual training*

In Figure 4A, mice initiated a new trial by licking the central spout, followed by a brief delay before entering the sample period. During the first 350ms of both the delay and sample period, the mice were required to refrain from licking the lateral spouts; doing so resulted in a noise cue and a timeout penalty. After the first 350ms of the sample period, the first lick on either lateral spout was recorded as the mouse’s choice for that trial, marking the end of the sample period. A correct choice resulted in 0.25μL water reward at the corresponding spout, while an incorrect choice triggered a noise cue and a timeout penalty. The stimuli presented during the sample period followed a specific sequence: sound frequency discrimination (3 kHz vs. 12 kHz), followed by reversed sound frequencies (12 kHz vs. 3 kHz), sound direction (left vs. right), reversed sound direction (right vs. left), and finally, light direction (left vs. right). Progression to the next stimulus condition occurred only when the mice achieved and maintained a performance accuracy above 75% in the previous discrimination task.

*DMS*

In Figure 4B, mice initiated a trial block by licking either the left or right spout. After a brief delay, they entered a 500ms sample period, during which a sound stimulus of either 3 kHz or 12 kHz was emitted from the top buzzer. Following this, a 1-second delay period began, during which licking either the left or right spout caused the trial to pause for 300ms. The delay period was followed by a 500ms test period, with another 3 kHz or 12 kHz sound stimulus delivered from the top buzzer. This was followed by a second 1-second delay period, during which the mice were again required to refrain from licking the lateral spouts. A brief response cue then indicated that the mice could express their choice by licking either the left or right spout. A correct choice resulted in 0.25μL water reward, while an incorrect choice led to a noise cue and a timeout penalty. When the sound frequencies presented during the sample and test periods were identical, the correct choice was the left spout. When the frequencies differed, the correct choice was the right spout. During the testing phase, the durations of the two delay periods were independently and randomly sampled from {1, 1.5, 2, 2.5, 3s}.

*d3AFC*

In Figure 4C, a paradigm similar to that described in Figure 2A was used, but with three possible choices. Mice could initiate a trial block by licking any of the three spouts. The task required them to discriminate between three different sound frequencies: 8 kHz, 16 kHz, and 32 kHz, with the corresponding spouts being left, center, and right, respectively, to receive a reward. During the testing phase, stimuli were logarithmically spaced around the 16 kHz midpoint, with values expanded to {-1, -0.75, -0.5, -0.25, 0, 0.25, 0.5, 0.75, 1}. Frequencies corresponding to {-1, -0.75, -0.5} indicated a left spout choice; {-0.25, 0, 0.25} indicated a center spout choice; and {0.5, 0.75, 1} indicated a right spout choice. In **Figure 3-figure supplement 1C**, for some mice that did not undergo the psychometric curve testing phase, the left and right stimulus-choice associations were reversed after they had learned the initial task, while the center stimulus-choice association remained unchanged. Whenever the mice achieved a 75% accuracy rate across all three trial types and maintained it for some time, the left-right stimulus-choice associations were immediately reversed again. Mice could only detect these reversals through trial feedback, with no additional cues indicating changes in task rules.

*Delayed Context-dependent task*

In Figure 4D, mice triggered a new trial by licking the central spout. After a brief delay, 500ms rule-indicating clicks stimulus was emitted from the top buzzer. This was followed by a delay period during which the mice were required to suppress licking the lateral spouts; otherwise, the current trial would end, accompanied by a noise cue and timeout penalty. Subsequently, 200ms auditory cue (3 kHz or 12 kHz) was delivered from either the left or right buzzer. Mice could report their choice 100ms into this cue. A correct choice resulted in 0.25μL water reward at the chosen spout, while an incorrect choice led to a noise cue and timeout penalty.

The click stimulus represented different task rules based on its frequency: 16 Hz clicks indicated that the mice should make their choice based on the direction of the sound, while 64 Hz clicks indicated that the reward was associated with the sound frequency. When the trial required attention to sound direction, the mice needed to choose based on the sound source location (left buzzer for left spout, right buzzer for right spout), ignoring the sound frequency. Conversely, for trials requiring attention to sound frequency, the mice had to discriminate the sound frequency (3 kHz for left, 12 kHz for right) to make their choice. The context and sample periods were separated by a 250ms delay period. Trials where the correct choices based on direction and frequency aligned were termed "coherent trials," while those where they conflicted were termed "conflict trials." Trials involving only one modality of stimulus were termed "unimodal trials" (for single sound direction trials, the frequency was set at 6 kHz; for single frequency trials, the sound was delivered from the top buzzer). Trials were randomly presented as 10% unimodal, 70% conflict, and 20% coherent, with mice required to achieve 75% accuracy on conflict trials.

During the testing phase, the delay duration was randomly chosen from a range of 200ms to 2000ms. Additionally, the click rate, representing the context, was extended to {16, 20, 25, 32, 40, 50, 64 Hz}, where rates below 32 Hz indicated attention to sound direction and rates above 32 Hz indicated attention to sound frequency. For trials with a 32 Hz click rate, the reward choice was randomly linked to either sound direction or frequency.
